# Engineering Bone-Mimetic Microspheres to Recapitulate the Tumor Microenvironment for In Vitro Osteosarcoma Modeling

**DOI:** 10.3390/biomedicines14040868

**Published:** 2026-04-10

**Authors:** Fangqiao Zheng, Zhengyi Lan, Hangrong Chen, Ming Ma

**Affiliations:** 1 School of Chemistry and Materials Science, Hangzhou Institute for Advanced Study, University of Chinese Academy of Sciences, Hangzhou 310024, China; 2 State Key Laboratory of High Performance Ceramics, Shanghai Institute of Ceramics, Chinese Academy of Sciences, Shanghai 200050, China

**Keywords:** osteosarcoma, droplet microfluidics, bone-mimetic microspheres, three-dimensional cell culture, in vitro tumor model, tumor microenvironment

## Abstract

**Background:** Osteosarcoma (OS) is an aggressive bone tumor. The lack of physiologically relevant three-dimensional models that recapitulate the native tumor microenvironment hampers drug development and mechanistic studies. The study aimed to develop bone-mimetic microspheres for the construction of an OS model. **Materials and Methods:** We employed droplet microfluidics to fabricate bone-mimetic microspheres (named MSHA) from a composite of gelatin methacryloyl, polyethylene glycol diacrylate, and nano-hydroxyapatite (nHA). MNNG/HOS cells were cultured on MSHA microspheres and subsequently evaluated for their bioactivity and capabilities of stemness, migration, and invasion. **Results:** The microfluidic platform enabled efficient and scalable production of highly uniform MSHA microspheres with controlled sizes. MNNG/HOS cells cultured on MSHA maintained high viability and spontaneously formed compact tumor spheroids after 7 days. Compared with two-dimensional cultures, cells cultured on these microsphere-based platforms exhibited enhanced migration and invasion capacities, along with increased expression of relevant biomarkers. RNA sequencing further revealed the activation of cancer-related pathways. Notably, the incorporation of nHA into microspheres amplified these malignant phenotypes, potentially through the activation of ECM–receptor interaction and calcium signaling pathways. **Conclusions:** The microfluidics-fabricated MSHA microspheres, as biomimetic three-dimensional culture scaffolds, offer a promising platform for applications in mechanistic studies of osteosarcoma progression and drug screening.

## 1. Introduction

Osteosarcoma (OS) is a malignant bone tumor mostly found in adolescents and young adults and is characterized by recurrence, metastasis, and poor prognosis [1]. However, progress in the treatment of OS has been limited [2,3]. The poor prognosis and survival of OS patients with the existing therapy techniques highlight an urgent need for novel therapeutic strategies [3,4]. Nonetheless, the development of new therapies is severely impeded by the lack of physiologically relevant in vitro models [5]. Currently, preclinical research into tumorigenesis and drug screening relies heavily on conventional two-dimensional (2D) cell cultures [6]. However, 2D culture fails to replicate the complexity of the in vivo tumor microenvironment and often leads to altered cellular phenotypes. Consequently, 2D models have limited predictive power for in vivo drug responses and tumor progression [7]. Remarkably, tissue-engineered 3D cancer models, through deliberate structural and compositional design, may more accurately recapitulate in vivo tumor cellular heterogeneity, morphology, and material transport [8,9]. They have now emerged as an ideal in vitro platform for OS research, offering a promising alternative to conventional 2D models.

The extracellular matrix (ECM) functions as both a structural scaffold and an active regulator of cancer progression, orchestrating tumor behavior and remodeling the tumor microenvironment [10]. Unlike soft tissue tumors, OS predominantly arises in the metaphyseal regions of long bones. This clinical feature underscores the pivotal role of the unique ECM in OS pathogenesis [11]. The ECM in OS is typically a stiff, mineralized organic–inorganic composite, in which type I collagen provides structural integrity and calcium phosphate imparts high mechanical modulus and osteoinductive signals [12]. These biophysical and biochemical cues are known to critically regulate tumor cell proliferation, invasion, and therapeutic resistance in OS [13]. In pursuit of more physiologically accurate and clinically predictive OS models, biomimetic 3D scaffolds combining nanohydroxyapatite (nHA) with collagen-based hydrogels have become a key strategy. For instance, Bassi et al. constructed a composite scaffold composed of magnesium-doped nHA and collagen fibers to mimic OS cancer stem cells niche. This bone-mimetic scaffold successfully preserved the stem-like phenotype of cancer stem cells [14]. In another study, González Díaz et al. fabricated a 3D OS model based on gelatin microribbons cross-linked with methacrylic anhydride and nHA. The model effectively simulated in vitro OS signaling pathways and drug response [15]. A key limitation of these scaffolds is their reliance on mold-casting, a low-throughput fabrication method that consequently hinders their use in large-scale investigations.

Droplet microfluidic technology has emerged as a high-throughput, automated, and integrated tool for the generation of 3D scaffolds [16,17]. This technology enables precise manipulation of micrometer-scale fluids, generating microspheres with highly tunable morphology and size for 3D cell culture applications [18]. Notably, compared with conventional tumor spheroid formation methods, the strategy of integrating droplet microfluidics with photopolymerization to fabricate hydrogel microspheres offers distinct advantages, including high batch-to-batch structural consistency and flexible material tunability. These features collectively enable more efficient and reliable high-throughput drug screening. Therefore, applying this integrated approach to engineer the OS ECM would combine the scalability of high-throughput fabrication with the biomimetic properties of tumor microspheres, thereby potentially accelerating the development and optimization of OS treatment strategies. However, studies specifically focused on this application remain scarce to date [19,20,21].

**Scheme 1:**
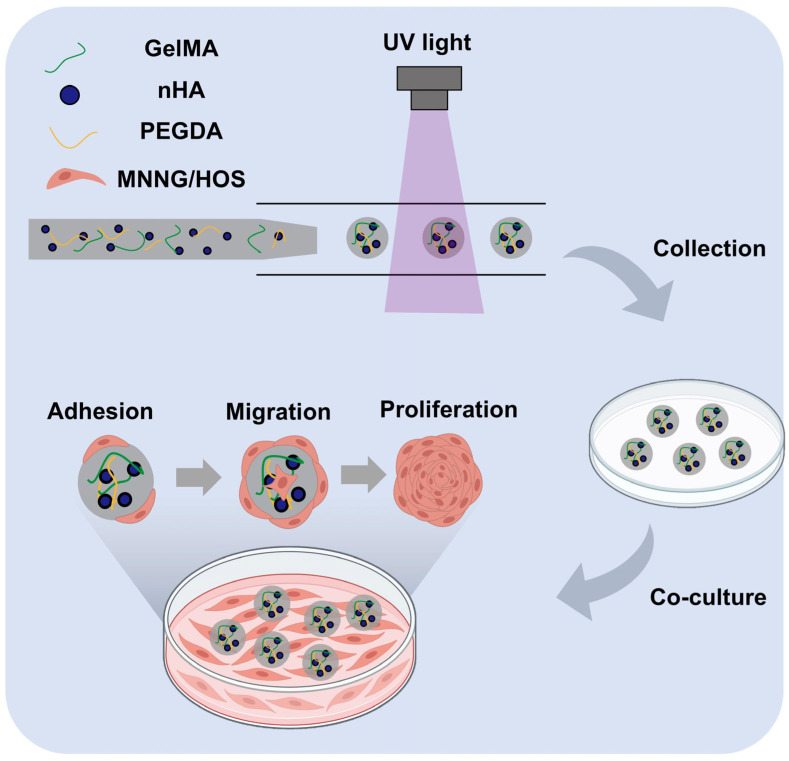
Schematic illustration of the preparation of bone-mimetic microspheres and their use as scaffolds for culturing MNNG/HOS cells. The aqueous precursor phase containing GelMA, PEGDA, nHA, and mineral oil were co-injected into the chip. Uniform water-in-oil droplets were generated at the junction, followed by in-line UV photocuring to form MSHA microspheres. MNNG/HOS cells were seeded onto the microspheres and cultured to form three-dimensional tumor spheroids.

This study aimed to develop bone-mimetic microspheres as an in vitro cell culture platform by employing a droplet-microfluidic synthesis strategy (Scheme 1). These microspheres were fabricated into micro-scaffolds using a composite of methacrylated gelatin (GelMA), polyethylene glycol diacrylate (PEGDA), and nHA, thereby recapitulating the biochemical composition of the native bone matrix. Through a series of functional assays, we evaluated the impact of the microsphere platform on MNNG/HOS cell stemness, migration, and invasion capacities, successfully achieving the goal of closely mimicking the biological behavior of clinical tumors. Beyond advancing fundamental research toward clinical translation, this platform, benefiting from its scalable production, provides an efficient experimental system for screening anti-tumor drugs.

## 2. Materials and Methods

### 2.1. Materials and Cell Lines

GelMA, PEGDA, and lithium phenyl (2,4,6-trimethylbenzoyl) phosphinate (LAP) were bought from Engineering for Life (Suzhou, China). nHA (H875580) and Span 80 were acquired from Macklin (Shanghai, China). Light mineral oil was purchased from Shanghai Rhawn Reagent Co., Ltd. (Shanghai, China). Collagenase type II was bought from Sigma-Aldrich (St. Louis, MO, USA). Cell Counting Kit-8 (CCK-8) was purchased from Dojindo Molecular Technologies, Inc. (Kumamoto, Japan). High-Glucose Dulbecco’s Modified Eagle’s Medium (HDMEM), PBS, Penicillin-Streptomycin, and trypsin were acquired from Shanghai BasalMedia (Shanghai, China). Calcein/PI Live/Dead Viability/Cytotoxicity Assay Kit and goat serum were bought from Beyotime Biotechnology (Shanghai, China). HE staining kit was bought from Shanghai Jingke Chemical Technology Co., Ltd. (Shanghai, China). TRIzol™ Reagent was purchased from Invitrogen (Carlsbad, CA, USA). RevertAid First Strand cDNA Synthesis Kit was bought from Fermentas (Vilnius, Lithuania). The SYBR Green PCR Kit was purchased from Applied Biosystems^TM^, Thermo Fisher Scientific (Foster City, CA, USA). The MNNG/HOS cell line was acquired from Shanghai Sixth People’s Hospital (Shanghai, China).

### 2.2. Design and Fabrication of Microfluidic Device

The microfluidic chip was constructed by a projection micro-stereolithography-based 3D printing technique (BMF Precision Tech Inc., Chongqing, China) with a high resolution of 10 µm. The microfluidic chip was designed by using the computer-aided design program SolidWorks 2022 (Dassault Systemes S.A.). The chip printing process was then carried out layer by layer, with each layer cured with UV light to build the complete structure.

### 2.3. Microfluidic Preparation of MSHA

A precursor solution was prepared by mixing 50.0 mg of GelMA, 50.0 μL of PEGDA, 20 mg of nHA (with a maximum diameter of less than 200 nm), and 25.0 mg of LAP in 1.0 mL of PBS. The incorporation of PEGDA ensures the long-term structural stability of the obtained microspheres. An oil phase was formulated by dissolving 5% (*w*/*w*) Span 80 in light mineral oil. To prepare the microspheres, the aqueous phase and oil phase were injected into the microfluidic chip by syringe pump (Lead Fluid (Baoding) Intelligent Equipment Manufacturing, Baoding, China) at different flow rates. A transparent silicone tube connected to the chip outlet served as a fixed irradiation zone. Droplets flowing through this tube were photocured by a UV lamp (395 nm, 10 W) and subsequently collected in PBS. The generated microspheres were centrifuged at 1000 rpm for 5 min, and the precipitate was collected and dispersed in PBS. The microspheres were finally dispersed in complete medium for further incubation. The resulting microspheres were termed MSHA (with nHA) and MS (without nHA). The morphology of MS and MSHA was observed using an optical microscope, followed by an evaluation of their size distribution.

### 2.4. Morphological, Mechanical, and Biodegradation Characterizations of MS and MSHA

The surface morphology and elemental mapping of the hydrogel microspheres were observed with a scanning electron microscope (SEM, Magellan 400, FEI Company, Hillsboro, OR, USA). Prior to SEM observation, the lyophilized samples were mounted on adhesive carbon tape and sputter-coated with gold to improve electrical conductivity [22]. To investigate the chemical bonding between nHA and the GelMA/PEGDA matrix, the freeze-dried nHA, MS, and MSHA samples were characterized by Fourier Transform Infrared Spectroscopy (FT-IR). The spectra were recorded over a range of 400–4000 cm^−1^ [23].

The compression performances of MS and MSHA were measured using a universal material testing machine (MTS E43.104, Eden Prairie, MN, USA). Test samples were prepared as cylinders (diameter: 8 mm; height: 8 mm) using the precursor solution. Three replicates were examined per hydrogel type. The compression speed was 1 mm min^−1^ [24]. The experiment was continued until mechanical failure was observed.

To evaluate the enzymatic degradability of the materials, 10.0 mg of freeze-dried microspheres were immersed in 500.0 µL of PBS containing 2 U mL^−1^ collagenase type II and incubated at 37 °C for 7 days [25,26]. The dissolution medium was refreshed daily to maintain consistent enzymatic activity. At the pre-determined time points, the PBS was removed. The MS and MSHA were washed three times with deionized water and then freeze-dried to measure residual weight. Three independent replicates were performed for each group at each time point (n = 3).

### 2.5. Biocompatibility of MSHA

The experimental procedures were consistent with those described in previous studies [25]. The only exception was that the cell seeding density was adjusted based on preliminary experiments. MNNG/HOS cells were grown for 7 days under three different conditions: (a) in standard 96-well plates for monoculture, (b) in 96-well low-attachment plates for co-culture with MS, and (c) in 96-well low-attachment plates for co-culture with MSHA. The experiments were performed with four replicates. Each well of the 96-well plates was seeded with 2 × 10^3^ MNNG/HOS cells. At the indicated time points (days 1, 4, and 7), CCK-8 reagent was supplemented into the culture medium at a final concentration of 10% (*v*/*v*). Aliquots (100 μL) of the culture medium taken from each well were pipetted into a fresh 96-well plate [26]. The absorbance at a wavelength of 450 nm was then determined using a microplate reader (BioTek, Winooski, VT, USA). The cell survival was then assessed using live/dead staining. The MSHA were photographed under a fluorescence microscope to capture the bright-field, red fluorescence, and green fluorescence images. The number of viable cells in each hydrogel microsphere was counted using ImageJ 1.54g software, with six microspheres randomly selected per condition for quantitative analysis.

### 2.6. Fluorescence Imaging and Immunofluorescence

The sample was washed three times with PBS and fixed with 4% paraformaldehyde at room temperature for 60 min. After three washes with PBS containing 0.4% Triton X-100, the sample was blocked at room temperature with PBS containing 10% goat serum. Samples were incubated overnight at 4 °C with antibodies against CD133 (1:200, 18495-1-AP, Proteintech, Wuhan, China), CD44 (1:200, 60224-1-LG, Proteintech, Wuhan, China), MMP9 (1:200, 30592-1-AP, Proteintech, Wuhan, China), Vimentin (1:200, AF0318, Beyotime, Shanghai, China), OPN (1:200, 22952-1-AP, Proteintech, Wuhan, China), and Ki67 (1:200, YA6300, MCE, Monmouth Junction, NJ, USA). This was followed by incubation with Alexa Fluor 594-conjugated anti-rabbit IgG H&L (1:200, HYP8003, MCE, USA) and Alexa Fluor 647-conjugated anti-mouse IgG H&L (1:200, HYP8101, MCE, USA) for 2 h at room temperature. Nuclear staining was achieved using DAPI (C1005, Beyotime, Shanghai, China). Cells were imaged using a confocal laser scanning microscope (FV1000, Olympus, Tokyo, Japan) [27].

### 2.7. RT-qPCR

Total RNA was extracted from 2D, MS, and MSHA group cultures using TRIzol^TM^ Reagent. The samples were mechanically homogenized in 1 mL of TRIzol^TM^ and then incubated for 15 min. RNA isolation was performed by the chloroform method and precipitated overnight with ethanol at 4 °C. The RNA pellet was resuspended in 50 μL of RNase-free water. For RT-qPCR, cDNA was synthesized from 1 μg of mRNA using the RevertAid First Strand cDNA Synthesis Kit. Real-time PCR was performed on an ABI Prism 7300 system (Applied Biosystems, Foster City, CA, USA) using the SYBR Green PCR Kit [25]. GAPDH served as the housekeeping gene, and relative gene expression levels were calculated using the 2^−ΔΔCt^ method, with internal normalization to GAPDH expression. Primer sequences used are shown in Table S1. Three independent replicates were analyzed.

## 3. Results

### 3.1. Microfluidic Synthesis of MSHA

**Figure 1:**
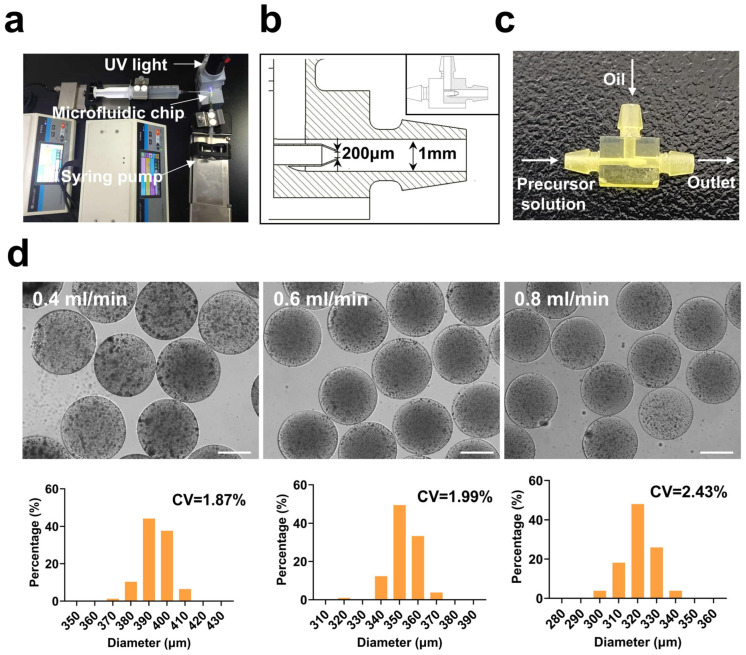
Fabrication of the MSHA using droplet microfluidics technology. (**a**) The microfluidic platform established for the generation of MSHA. (**b**) The cross-sectional diagram of the microfluidic chip fabricated by 3D printing (printing resolution: 10 μm). (**c**) The physical photograph of the microfluidic chip fabricated by 3D printing. (**d**) Optical microscopy images of MSHA synthesized at different oil phase flow rates (0.4, 0.6, and 0.8 mL/min) with a constant aqueous phase flow rate of 20 μL/min (scale bar: 200 μm). The corresponding particle size distribution statistics are shown below, with coefficient of variation (CV) values of 1.87%, 1.99%, and 2.43% (The randomly counted number of microspheres is 60).

The MSHA were constructed using a co-flow microfluidic chip, as illustrated in the schematic. Specifically, a custom-developed co-flow microfluidic chip with defined geometry and dimensions was fabricated using a 3D printing method based on projection micro-stereolithography, achieving a printing accuracy of 10 µm (Figure 1a). The outlet diameter of the intermediate phase channel of the microfluidic chip was 200 µm, while the outer phase channel diameter was 1000 µm (Figure 1b,c). This high-precision printing technique enabled precise control over channel dimensions, which is essential for the batch synthesis of droplets that meet the required size accuracy.

During the droplet generation process, the aqueous precursor solution was pumped into the intermediate phase channel, while the oil phase was injected into the outer channel. Uniform microdroplets were subsequently formed one by one at the junction of the co-flow structure due to the shear force exerted by the oil on the precursor solution. The generated microdroplets were then immediately subjected to UV curing to form MSHA with enhanced structural stability. We investigated the effect of the oil phase flow rate on droplet generation by increasing it from 0.4 to 0.8 mL/min while keeping the aqueous phase flow rate constant at 20 μL/min. The results showed that as the oil phase flow rate increased, the diameter of the resulting microspheres gradually decreased from approximately 390 to 320 µm. This result is in agreement with the well-established trend in droplet microfluidics, where higher flow rates of the continuous phase increase the shearing force, thereby producing smaller droplets [28]. More importantly, regardless of the oil phase flow rate adjusted, the MSHA products obtained at each flow rate maintained good batch-to-batch uniformity (CV < 3%), as shown in Figure 1d. The condition with the lowest CV (1.87%), corresponding to an oil phase flow rate of 0.4 mL/min, was selected for subsequent experiments. Under this condition, the microsphere generation rate reached 600 microspheres per minute. In summary, the combination of high morphological uniformity and scalable production makes this microfluidic platform particularly suitable for generating standardized tumor microsphere models, which are essential for reproducible and high-throughput drug screening applications.

### 3.2. Physicochemical Properties of MSHA

**Figure 2:**
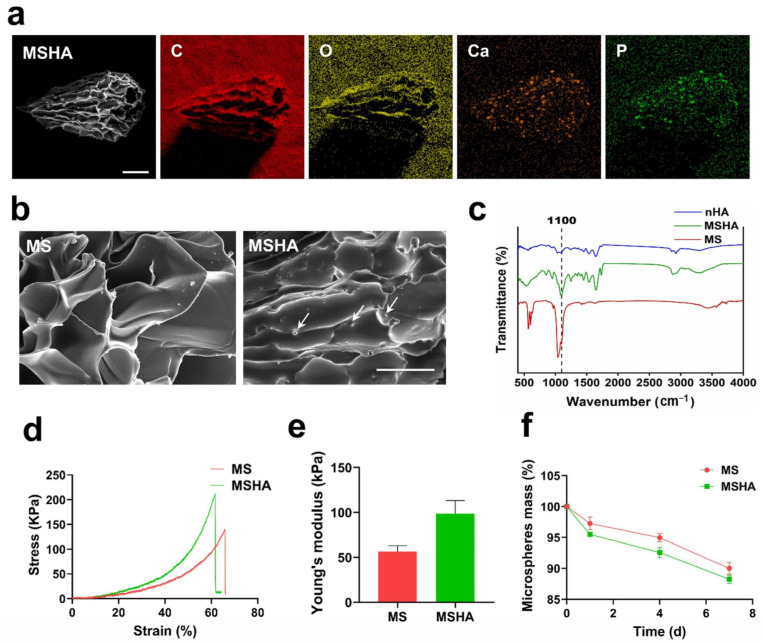
Microstructural and mechanical properties characterization of MS and MSHA. (**a**) SEM images and EDS elemental mapping of C, O, Ca, and P in MSHA. Scale bar: 250 μm. (**b**) SEM image of the randomly selected scaffold surface of MS and MSHA. Arrows in the figure indicate the position of nHA particles. Scale bar: 50 μm. (**c**) FTIR spectra of nHA, MS, and MSHA recorded over a range of 400–4000 cm^−1^. (**d**) Representative compressive stress–strain curve of MS and MSHA (n = 3). (**e**) Young’s modulus of MS and MSHA calculated from the linear elastic region of the stress–strain curves (n = 3). (**f**) Enzymatic degradation profile of MS and MSHA in PBS containing 2 U mL^−1^ collagenase type II over 7 days (n = 3 per time point).

The microstructural characterization of MSHA was conducted to assess their utility as bone-mimetic scaffolds for 3D tumor culture. First, scanning electron microscopy (SEM) imaging and energy-dispersive X-ray spectroscopy (EDS) elemental mapping were performed on randomly selected, freeze-dried MSHA samples (Figure 2a and Figure S1). The SEM and EDS results demonstrated that the nHA was well-distributed inside the MSHA. In addition, SEM of the MSHA surface revealed that the incorporation of nHA rendered the microspheres rough (Figure 2b). The pore size of the microspheres is approximately 12.1 μm (Figure S2). Infrared spectroscopy was performed on nHA, MS, and MSHA, respectively, as shown in Figure 2c. The C-O stretching vibration peak at 1100 cm^−1^ of the strong hydroxyl group in the MS hydrogel was weakened by the addition of nHA. This enhancement may be attributed to the formation of [HO]-Ca^2+^-[OH] bridging structures, where Ca^2+^ originating from nHA coordinates with the -OH groups on the GelMA/PEGDA network, thereby integrating the components through hydrogen bonding [29].

Compared with MS, the MSHA exhibited increased maximum compressive stress, but reduced maximum compressive strain (Figure 2d). At the same time, Young’s modulus significantly increased, from 56.6 kPa in the original MS hydrogel to 98.6 kPa in the MSHA composite, indicating that the addition of nHA makes the hydrogel network stiffer and stronger, but less deformable (Figure 2e).

Furthermore, the degradation profiles of MS and MSHA were evaluated. Specifically, MS degraded more rapidly (11.8%) than MSHA (9.7%) after 7 days. MSHA displayed a relatively slow degradation rate and maintained a clear scaffold morphology in an enzymatic environment (Figure 2f and Figure S3). Collectively, these results indicate that the MSHA possessed a robust and stable 3D network structure, which can support long-term 3D cell culture.

### 3.3. Biocompatibility and Spheroid-Promoting Capability of MSHA

**Figure 3:**
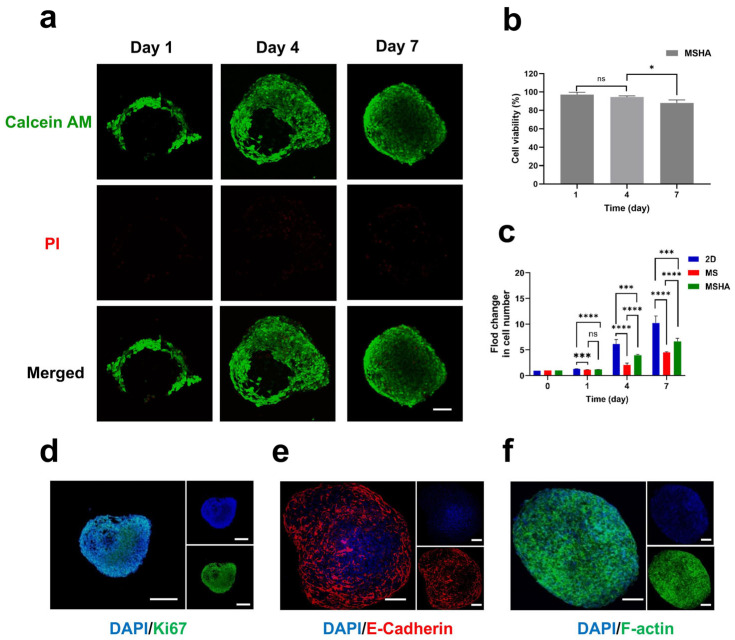
Biocompatibility and spheroid formation capability of MSHA. (**a**) Live/dead staining of MNNG/HOS cells cultured on MSHA. Viable cells were stained with Calcein-AM (green) and dead cells with propidium iodide (red). Scale bar: 100 μm. (**b**) Percentage viability of MNNG/HOS cells co-cultured with MSHA on days 1, 4, and 7, quantified from live/dead staining images (n = 6). (**c**) Quantification of the viability of MNNG/HOS cells cultured on 2D plates, MS, and MSHA on days 1, 4, and 7 using the CCK-8 assay (n = 4). (**d**–**f**) Fluorescence images of MNNG/HOS spheroids in MSHA stained with DAPI (blue) and Ki-67 (**d**), E-cadherin (**e**), or phalloidin (**f**). The scale bar for (**d**) is 200 μm, and the scale bars for (**e**,**f**) are 100 μm. * *p* < 0.05, *** *p* < 0.001, and **** *p* < 0.0001; ns, not significant.

The components of MSHA, including PEGDA, GelMA, and nHA, exhibit excellent biocompatibility and are widely used for tissue regeneration. Therefore, the MSHA were expected to demonstrate a similar safety profile. To generate MNNG/HOS cell-loaded MSHA, MNNG/HOS cells and MSHA were co-incubated in 6-well plates with ultra-low attachment surfaces. Given that MSHA provided adhesion sites for MNNG/HOS cells, the cells spontaneously adhered to the microspheres and migrated, as confirmed by the distribution of viable green fluorescent cells following live/dead staining (Figure 3a). Furthermore, negligible red fluorescent (necrotic) cells were observed in the live/dead staining images, indicating that MSHA exerted no significant cytotoxic effects on MNNG/HOS cell viability (Figure 3b).

We further compared the proliferation of MNNG/HOS cells cultured on 2D plates, MS, and MSHA over time. After four days of culture, cells in 3D culture exhibited a slower proliferation rate compared to those in 2D culture. At the same time, nHA can promote the proliferation of MNNG/HOS cells on the surface of the microspheres (Figure 3c). We further characterize the cell cycle status of OS cells under different culture conditions (Figure S4). PI staining confirmed that 3D culture significantly increased the proportion of G1-phase cells while reducing those in S and G2 phases, indicating obvious G1 arrest in MNNG/HOS cells. No significant difference in cell cycle distribution was observed between the MS and MSHA groups on day 7, which can be attributed to the formation of dense and mature spheroids in the MSHA group. Western blot results revealed decreased expression of P-Rb and CDK4 as well as elevated p53 in 3D-cultured cells (Figure S5), which was consistent with the G1 arrest phenotype. Notably, ANKRD2 was markedly upregulated in the 3D groups, further supporting the regulatory effect of the 3D microenvironment on cell cycle progression in MNNG/HOS cells. The increased expression of ANKRD2 may be attributed to oxidative stress induced by the 3D culture microenvironment [30].

Furthermore, cells on the MSHA surface were stained with an antibody against Ki-67, a well-established proliferation marker expressed in proliferating cells and associated with ribosomal RNA transcription [31]. The presence of Ki-67-positive cells after seven days of culture further indicated that MSHA supported long-term active proliferation (Figure 3d). Notably, after seven days of culture, the MNNG/HOS cell-loaded MSHA had condensed into compact spheroids (Figure S6). The distinct expression of E-cadherin (a key transmembrane protein mediating homophilic cell–cell adhesion) and the organized distribution of the actin cytoskeleton further confirmed spheroid formation (Figure 3e,f). Collectively, these results demonstrate that MSHA provided a biocompatible and supportive scaffold for the long-term 3D culture of OS cells, promoting cell adhesion, proliferation, and self-organization into compact spheroids.

### 3.4. Stemness, Migration, and Invasion of MNNG/HOS Cells on MS and MSHA

**Figure 4:**
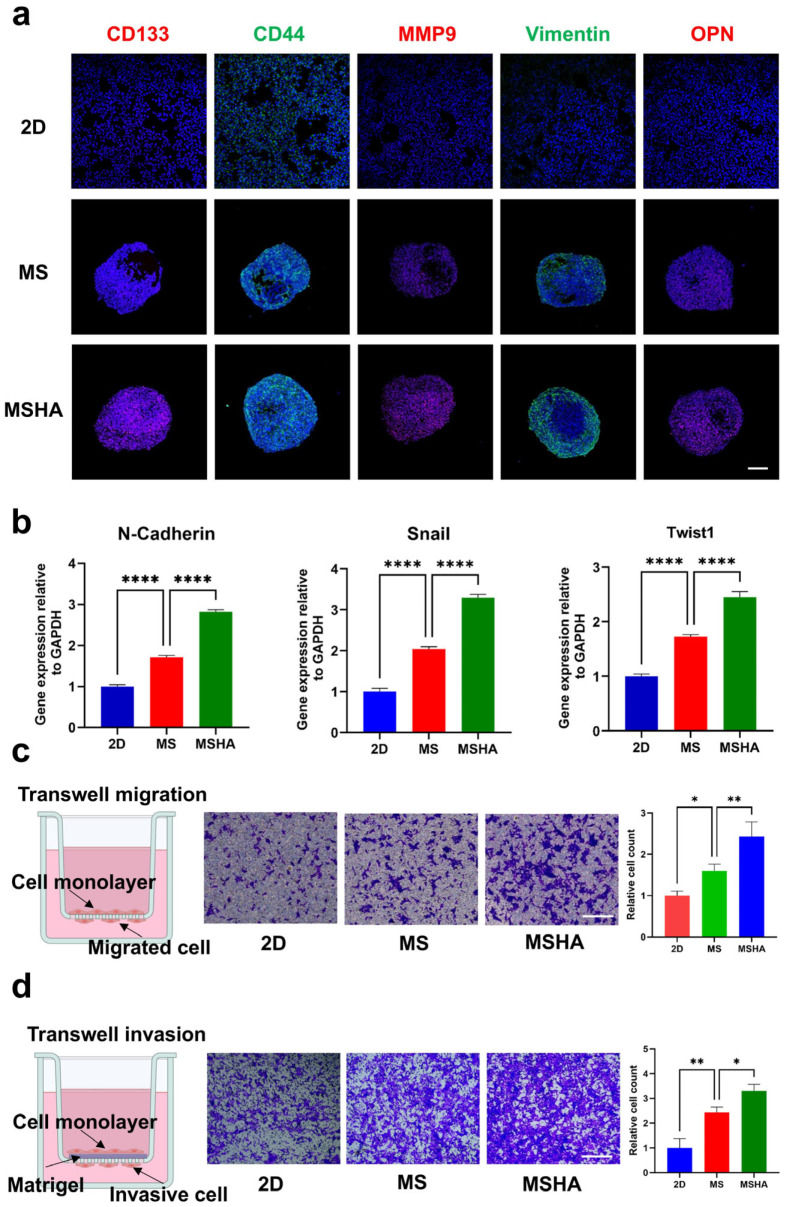
Characterization of stemness, invasion potential, and migratory ability of MNNG/HOS cells cultured on MS and MSHA. (**a**) Immunofluorescence images showing the expression of CD133, CD44, MMP9, Vimentin, and OPN in MNNG/HOS cells cultured on 2D, MS, and MSHA. Scale bar: 100 μm. (**b**) Relative mRNA expression levels of EMT-related genes (N-cadherin, Snail, Twist1) determined by RT-qPCR (n = 3). (**c**) Transwell assay and quantitative analysis of the migratory ability of MNNG/HOS cells cultured on 2D, MS, and MSHA (n = 3). Scale bar: 100 μm. (**d**) Transwell assay and quantitative analysis of the invasive capability of MNNG/HOS cells cultured on 2D, MS, and MSHA (n = 3). Scale bar: 200 μm. * *p* < 0.05, ** *p* < 0.01, and **** *p* < 0.0001.

Immunofluorescence analysis was employed to compare the expression of stemness and metastasis-associated proteins in MNNG/HOS cells cultured under 2D, MS, and MSHA conditions. As shown in Figure 4a, the protein expression of CD133 and CD44 was significantly higher in MS and MSHA groups than in 2D, with the highest levels in MSHA (MSHA > MS > 2D). This finding is consistent with accumulating evidence that tumor cells cultured in 3D systems exhibit enhanced stemness properties and self-renewal capacity. Moreover, upregulation of MMP9, vimentin, and OPN in MS and MSHA groups reflects a more invasive phenotype, again with the highest levels in MSHA. Western blot analysis further validated the expression trends of CD133, CD44, MMP9, vimentin, and OPN, showing the same changing pattern across the three culture conditions as observed by immunofluorescence (Figure S7). Collectively, these data indicate that MS and MSHA scaffolds promote an aggressive tumor phenotype, with MSHA exerting a stronger effect.

Consistently, the mRNA expression levels of N-cadherin, Snail, and Twist1 were significantly elevated in MNNG/HOS cells cultured in MS and MSHA (Figure 4b). Relative to 2D plate cultures, the mRNA levels of N-cadherin in the MS group increased by 1.7-fold, respectively, while this in the MSHA group showed increases of 2.8-fold, respectively; Snail levels were elevated by 1.7-fold in the MS group, and by 2.5-fold in the MSHA group; and Twist1 levels were upregulated by 2.0-fold in the MS group, and by 3.3-fold in the MSHA group. The findings indicate that both MS and MSHA scaffolds effectively promote epithelial–mesenchymal transition (EMT) in MNNG/HOS cells, positioning them as promising in vitro models for investigating drug resistance mechanisms and for related drug screening applications.

To further evaluate functional behavior, Transwell migration and invasion assays were performed on all three cell groups. The results demonstrated that, compared to the 2D culture condition, both the MS and MSHA scaffolds enhanced migration and invasion, with the MSHA group displaying the highest migratory and invasive capacities, followed by the MS group (MSHA > MS > 2D) (Figure 4c,d). The increases were approximately 1.9-fold and 2.4-fold for migration and 2.5-fold and 3.3-fold for invasion in the MS and MSHA groups, respectively, relative to the 2D control.

### 3.5. Genome-Wide Gene Expression of MNNG/HOS Cells Cultured in 2D, MS, and MSHA Group

**Figure 5:**
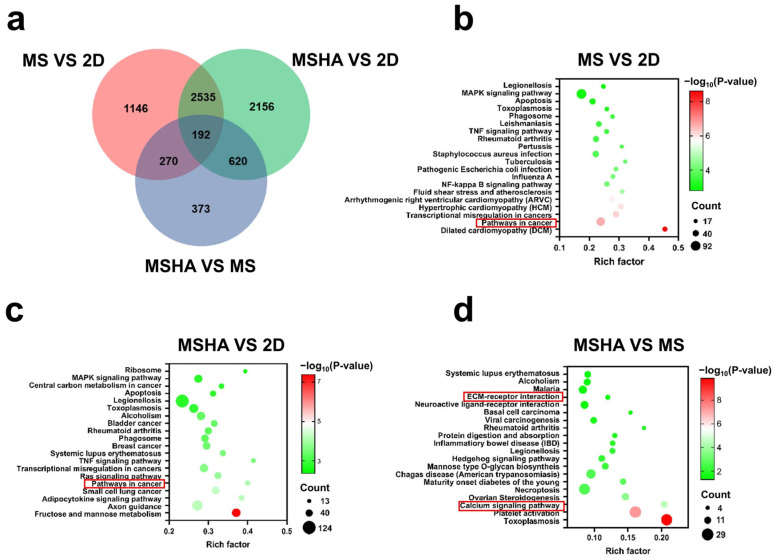
Global transcriptomic differences between the 2D monolayer and MSHA models. (**a**) Venn diagram of differentially expressed genes (MS vs. 2D, MSHA vs. 2D, MSHA vs. MS). DEGs were defined as |log_2_ (Fold Change)| ≥ 1 and adjusted *p* < 0.05. (**b**) KEGG enrichment analysis of MS vs. 2D (top 20 enriched pathways). Pathways of interest are highlighted in red. (**c**) KEGG enrichment analysis of MSHA vs. 2D. (**d**) KEGG enrichment analysis of MSHA vs. MS.

To elucidate global transcriptomic differences among 2D, MS, and MSHA cultures, we performed RNA-seq on MNNG/HOS cells cultured from each condition. Comparative analysis revealed 2410 upregulated and 3093 downregulated genes in the comparison of MS and 2D culture, while 1522 genes upregulated and 2621 genes downregulated in the comparison of MSHA with 2D culture (Figure 5a). KEGG pathway enrichment analysis identified the top 20 enriched pathways, among which “pathways in cancer” were significantly altered (Figure 5b,c). Pathways in cancer is a complex pathway that encompasses the regulation of multiple tumor-related pathways [32]. The significantly differentially expressed genes in MSHA vs. 2D (e.g., PIK3R2, AKT1, VEGFB, FOS, MDM2, BBC3, PMAIP1, GADD45A, STK4, EGLN3, SLC2A1, PIM2) encode multiple cell signaling factors. The expression of activators involved in several other crucial pathways was also found to be significantly altered in the 3D scaffold-based OS model: MDM2, BBC3, PMAP1, GADD45A in p53 signaling pathway, PIK3R2, AKT1, and VEGFB in the PI3K/Akt signaling pathway, FOS in the MAPK signaling pathway, PIM2 in the JAK/STAT signaling pathway, STK4 in the hippo signaling pathway, and EGLN3 and SLC2A1 in the HIF signaling pathway (Figure S8). RT-qPCR further validated the expression trends of these genes (Figure S9). These findings demonstrated that MNNG/HOS cells cultured in scaffolds (MS and MSHA) exhibited concurrent activation of multiple signaling pathways. Furthermore, KEGG enrichment analysis of MSHA vs. MS revealed that ECM–receptor interaction and calcium signaling pathways were significantly altered (Figure 5d). Consistently, GSEA confirmed significant upregulation of these two pathways in the MSHA model (Figure S10).

## 4. Discussion

Biomimetic three-dimensional OS models represent a critical step forward in preclinical research and drug screening. There is growing recognition that the tumor niche plays a pivotal role in cancer progression. The physical microenvironment of OS encompasses both trabecular and cortical bone, as well as the bone marrow compartment [33]. Current OS model research increasingly focuses on the construction of bone-mimetic scaffolds to replicate its unique tumor niche. However, most existing scaffolds are relatively large in size and are often constructed by mold-casting or 3D printing, limiting their applicability in large-scale drug screening [34,35,36]. Therefore, there is an urgent need to develop a bone-mimetic matrix scaffold suitable for high-throughput, rapid construction to effectively establish an in vitro model of OS. In this study, we developed bone-mimetic microspheres as micro-scaffolds for OS model construction. Droplet microfluidic technology was employed as the fabrication technique for its capacity to enable high-throughput production of uniform and monodisperse microspheres. The results indicate that the bone-mimetic microspheres effectively supported tumor cell adhesion and proliferation, and then formed compact tumor spheroids. The bone-mimetic microspheres were essential for the maintenance of cancer stem cells, enhanced the capabilities of migration and invasion, and the related EMT markers for OS, which aligned with previous research [25].

In terms of material composition, GelMA, PEGDA, and nHA are typically applied in the construction of a bone scaffold [23]. GelMA can be prepared by linking the methacrylate groups to soluble proteins derived from the partial hydrolysis of collagen [37]. It retains the cell-binding sequence RGD from degraded collagen, thereby serving as a common substitute for tissue collagen. The RGD groups of the GelMA effectively facilitate cell adhesion to the scaffold by interacting with integrin receptors on the cell surface [38]. PEGDA is a synthetic biocompatible material commonly used in cell culture. The integration of PEGDA into GelMA hydrogel demonstrates enhanced mechanical properties, making it suitable for prolonged cell studies [39]. For instance, the degradation rate of MS (11.8% at 7 days) was lower than that reported for pure GelMA microspheres in OS culture models. nHA was further incorporated to provide mineral cues analogous to the inorganic phase of bone. Its presence contributed to additional mechanical reinforcement and stabilization, as evidenced by the reduced weight loss of MSHA (9.7% at 7 days) compared to MS, as well as the increased Young’s modulus from 56.6 kPa to 98.6 kPa [39]. FTIR analysis confirmed interfacial interactions between nHA and the polymer matrix through [HO]–Ca^2+^-[OH] bridging structures, which likely underpin the observed mechanical enhancement. This finding is consistent with previous studies reporting similar coordination interactions between hydroxyapatite and polymer networks [23]. The exceptional biocompatibility and bioactivity of nHA are attributed to its structural and compositional similarity to the mineral component of human bone [40]. Therefore, microspheres cause no damage to cell viability. Furthermore, research has revealed that nHA supports retention of OS signaling and drug resistance, and enhances surface roughness by increasing cell adhesion sites [15]. Therefore, when co-cultured with the MSHA microspheres, tumor cells adhered to the microsphere surface, exhibiting higher proliferative activity compared to that co-culture with MS. However, the proliferation rate of cells on the scaffolds was lower than that in 2D culture. The decrease in the proliferation rate of cells on microspheres may be attributed to contact inhibition and the physicochemical cues of deposited ECM [41]. Notably, these growth kinetics more closely mimic in vivo tumor proliferation.

3D scaffold-supported OS models have been shown to exhibit enhanced stemness, migration, and invasion due to their faithful recapitulation of the in vivo tumor microenvironment [42]. MNNG/HOS cells cultured on scaffold (MS and MSHA) showed a significant increase in the cancer stem cell markers CD133 and CD44, the matrix-remodeling enzyme MMP9, the cytoskeletal protein Vimentin, and the multifunctional cytokine OPN. CD133 enhances tumor migration and metastatic capacity by inducing EMT and activating downstream signaling pathways such as Src-FAK, EGFR-Akt, and NF-κB [43]. Similarly, CD44 acts as a key driver molecule in cancer development and progression by interacting with the extracellular matrix, resulting in EMT and angiogenesis [44]. MMP9 is an enzyme that mediates extracellular matrix degradation and bone remodeling. This enzyme is frequently dysregulated in OS and promotes tumor invasion and metastasis [45]. Vimentin, a key intermediate filament protein, contributes to cell migration by dismantling apical–basal polarity and restructuring the cytoskeleton [46]. OPN enhances tumor migration, invasion, and drug resistance by inducing EMT and activating key tumor-related signaling pathways [47]. The observed upregulation of markers mentioned above in the MS and MSHA model may be attributed to activation of multiple tumor pathways.

Furthermore, the incorporation of nHA appeared to enhance tumor metastasis and invasion, potentially by modulating the ECM and regulating calcium ions. Specifically, nHA promoted the formation of compact, ECM-enriched tumor spheroids on the microsphere surface. We hypothesize that the subsequent interaction between these ECM macromolecules and cellular integrins mediates cell adhesion. This interaction, in turn, activates bidirectional signaling and mechanotransduction, ultimately regulating the cellular signaling network to facilitate invasion [32]. Accumulating evidence establishes that dysregulated calcium signaling drives tumor initiation and progression. As an essential second messenger, calcium modulates key signaling pathways involved in tumor metastasis. Moreover, transient calcium accumulation facilitates tumor metastatic progression, with calcium-dependent proteins and calcium-related channels contributing substantially to this process [48].

Consistently, many signaling transduction molecules showed significant upregulation in MSHA, further supporting the notion that the MSHA model effectively recapitulates the complex signaling crosstalk inherent to the OS TME. Importantly, EMT has been intimately linked to drug resistance in multiple cancer types, including OS. Tumor cells undergoing EMT acquire resistance to conventional chemotherapeutics and targeted agents through multiple mechanisms, including enhanced drug efflux, evasion of apoptosis, and adoption of stem-like properties [49]. Therefore, MSHA could be a promising platform for studying the molecular mechanisms of OS progression and developing therapeutic strategies to overcome drug resistance.

However, this study has several limitations. Firstly, considering the physiological stiffness of the bone matrix (0.1–20 GPa), future research should focus on increasing the mechanical stiffness of the inks to more faithfully mimic the OS-TME [50]. Increasing the PEGDA and nHA concentrations offers a viable strategy to enhance the compressive modulus of GelMA scaffolds. Another approach entails the implementation of post-treatment, wherein mineralization is utilized to enhance gel strength [51]. Secondly, further validation in stromal cell-involved systems is essential for findings from in vitro scaffold-based OS models, owing to the highly heterogeneous nature of tumor tissue [9]. Future investigations should explore the feasibility of integrating this microsphere-based platform with co-culture systems to better mimic the cellular complexity of the OS niche. Despite these limitations, this study identifies promising directions for further refinement and application of the MSHA model.

## 5. Conclusions

This study developed highly uniform, bone-mimetic microspheres via a microfluidic technology, and employed them to establish a 3D OS culture model. MNNG/HOS cells cultured on these microspheres self-assembled into compact tumor spheroids. Compared with conventional 2D culture, cells grown on the microspheres exhibited significantly enhanced stemness, invasiveness, and migration potential, thereby more closely recapitulating the malignant phenotype observed in vivo. Specifically, the incorporation of nHA into the microspheres not only promotes cell adhesion by enhancing ECM deposition but also activates calcium signaling pathways, both of which are recognized as key drivers of MNNG/HOS cell migration and invasion. This platform overcomes key limitations of traditional bone-mimetic scaffolds, such as poor uniformity and low production efficiency, providing a reliable and scalable in vitro model for mechanism studies of OS and high-throughput screening of antitumor compounds.

## Data Availability

The original contributions presented in this study are included in the article/Supplementary Materials. Further inquiries can be directed to the corresponding author.

## Supplementary Materials

The following supporting information can be downloaded at: https://www.mdpi.com/article/10.3390/biomedicines14040868/s1.
