# Supplementary material for: Engineering Bone-Mimetic Microspheres to Recapitulate the Tumor Microenvironment for In Vitro Osteosarcoma Modeling
Source: Biomedicines. 2026 Apr 10;14(4):868. doi: 10.3390/biomedicines14040868 (PMC13113478; doi:10.3390/biomedicines14040868)
Supplement: Supplementary file 1 [file biomedicines-14-00868-s001.zip › biomedicines-4208218-supplementary.pdf]

Supporting Information

Engineering Bone-Mimetic Microspheres to Recapitulate the Tumor Microenvironment for In Vitro Osteosarcoma Modeling

*Fangqiao Zheng[1, a], Zhengyi Lan[2, a], Hangrong Chen[1, 2], Ming Ma[1, 2, *]*

[1]School of Chemistry and Materials Science, Hangzhou Institute for Advanced Study, University of Chinese Academy of Sciences, Hangzhou 310024, China

[2] State Key Laboratory of High Performance Ceramics, Shanghai Institute of Ceramics, Chinese Academy of Sciences, Shanghai 200050, P. R. China

[a] These authors contributed equally to this work

[*] Corresponding author: Prof. Ming Ma (Email: mma@mail.sic.ac.cn)

Supplementary Figures and Tables

Figure S1. EDS spectrum with quantitative result.

Figure S2. Pore size distribution of MSHA. Scale bar: 20 μm.

Figure S3. Optical microscopic image of MSHA after 7 days in an enzymatic environment. Scale bar:100 μm.

Figure S4. Cell cycle and quantitative results of MNNG/HOS cells by FC.

Figure S5. Protein expression levels of p53, Phospho-Rb (P-Rb), CDK4, and ANKRD2 in the 2D, MS, and MSHA groups.

Figure S6. Optical microscopic image of a spheroid based on MSHA. Scale bar: 200 μm.

Figure S7. Protein expression of CD133, CD44, MMP9, Vimentin, and OPN, which are related to cell stemness, invasion, and migration.

Figure S8. Expression of signaling transduction factors of pathways in cancer.

Figure S9. Gene expression of signal transduction factors involved in cancer-related pathways, as assessed by RT-qPCR.

Figure S10. GSEA analysis of significantly changed pathways.

Experimental apparatus and methods

Cell culture

MNNG/HOS cells were cultured in HDMEM, supplemented with 10% v/v fetal bovine serum, 100 Unit/mL penicillin, and 100 μg/mL streptomycin. Cells were cultured in T-25 flasks or petri dishes for cell reproduction at 37°C and 5% CO_2 in an incubator.

Transwell migration and invasion assays

For migration assay, MNNG/HOS cells derived from 2D, MS, and MSHA cultures were seeded into the upper chambers of Transwell inserts (12 μm pore size) and cultured for 24 h in serum-free medium, with complete medium in the lower chambers. For invasion assay, the upper chambers were pre-coated with Matrigel before cell seeding. Migrated and invaded cells were stained with crystal violet and visualized under a light microscope. Migration and invasion assays were performed in triplicate experiments[1].

Flow cytometry

MNNG/HOS cells were cultured for seven days, then harvested and stained with cell cycle detection kits (beyotime, China). The results were analyzed by flow cytometer (BD LSR Fortessa). Three experiments were conducted[1].

Western blot

MNNG/HOS cells were lysed using RIPA buffer (Thermo Fisher Scientific). Protein concentrations were measured via the Pierce BCA Protein Assay (Thermo Fisher Scientific). Equal amounts of cell extracts were loaded into NuPAGE Bis-Tris Protein Gels for electrophoresis. Protein bands were transferred onto PVDF membranes and subsequently blocked in 10% BSA and incubated with the following antibodies at 4°C overnight: p53 (1:500, abcam, ab131442), Phospho-Rb (1:1000, abcam, ab184796), CDK4 (1:1000, CST, #88428), ANKRD2 (1:1000, ptglab, 11821-1-AP), CD133 (1:1000, CST, #64326), CD44 (1:1000, abcam, ab189524), MMP9 (1:500, genetex, GTX100458), Vimentin (1:1000, genetex, GTX636980), osteopontin (OPN) (1:500, affbiotech, AF0227). β-actin (1:1500, abcam, ab8226) was used as a loading control. Following primary antibody incubation, membranes were incubated in HRP (1:1000,

Beyotime, A0208) or anti-mouse HRP (1:1000, Santa Cruz Biotechnology) secondary antibody for 1 h at room temperature. Membranes were then incubated in SuperSignal^{TM} West Femto Maximum Sensitivity Chemiluminescent Substrate (Thermo Scientific, cat. 34095) for band visualization.

RNA sequencing

Total RNA was extracted from MNNG/HOS cells cultured in 2D, MS, and MSHA using TRIzol reagent. A cDNA library was then constructed with the TruSeq Stranded mRNA Sample Preparation Kit. Sequencing was carried out on the Illumina NovaSeq 6000 platform, with three biological replicates per group. Raw sequencing reads were quality-trimmed using Trimmomatic software. Differential gene expression analysis was conducted with the DESeq2 R package, followed by pathway analysis of Kyoto Encyclopedia of Genes and Genomes (KEGG) and Gene Set Enrichment Analysis (GSEA)[2]. The experiment was performed in triplicate.

Statistical analysis

Data were analyzed using GraphPad Prism 9.0 and IBM SPSS Statistics v.23.0. An unpaired two-tailed Student's t-test was performed for statistical comparisons. All values are presented as mean ± S.D. with indicated sample size. Statistical significance was defined as $*p < 0.05$, $**p < 0.01$, $***p < 0.001$, and $****p < 0.0001$; ns, not significant.

Supplementary Figures and Tables

Primer Name	Primer Sequences
N-cadherin-F	5' CATCATCCTGCTTATCCTTG 3'
N-cadherin-R	5' AAGTCATAGTCCTGGTCTTC 3'
Snail-F	5' TTCCTGAGCTGGCCTGTCTG 3'
Snail-R	5' TGGCCTGAGGGTTCCTTGTG 3'
Twist1-F	5' AGTCCGCAGTCTTACGAG 3'
Twist1-R	5' GCTTGCCATCTTGGAGTC 3'
PIK3R2-F	5' GAAAGGCGGGAACAATAAG 3'
PIK3R2-R	5' ATTTGGACACAGGGTAGAG 3'
AKT1-F	5' CTACAACCAGGACCATGAGAAG 3'
AKT1-R	5' ACACGATACCGGCAAAGAAG 3'
VEGFB-F	5' TGTGTATACTCGCGCTACC 3'
VEGFB-R	5' ACCGGATCATGAGGATCTG 3'
FOS-F	5' AAGCGGAGACAGACCAAC 3'
FOS-R	5' TCAAGGGAAGCCACAGAC 3'
MDM2-F	5' TCCTGAAGATAAAGGGAAAG 3'
MDM2-R	5' AAGTTGATGGCTGAGAATAG 3'
BCL2-F	5' AGATGGAGCCCAATTAGG 3'
BCL2-R	5' CATGGTGCAGAGAAAGTC 3'
PMAIP1-F	5' AGCTGGAAGTCGAGTGTGCTAC 3'
PMAIP1-R	5' TCAGGTTCCTGAGCAGAAGAG 3'
GADD45A-F	5' CCCGATAACGTGGTGTTG 3'
GADD45A-R	5' GCGCAGGATGTTGATGTC 3'
SKT4-F	5' AAATCCTCCTCCCACATTCC 3'
SKT4-R	5' GGCGTTTCAGTTTCACATCC 3'
EGLN3-F	5' GCTTGCTATCCGGGAAATG 3'
EGLN3-R	5' TGTGGGTTCCTACGATCTG 3'
SLC2A1-F	5' CAGATGATGCGGGAGAAGAAG 3'

SLC2A1-R	5' ACAAACAGCGACACGACAG 3'
PIM2-F	5' AATCCCATCACCATCTTC 3'
PIM2-R	5' AGGCTGTTGTCATACTTC 3'
GAPDH-F	5' CACCCACTCCTCCACCTTTG 3'
GAPDH-R	5' CCACCACCCTGTTGCTGTAG 3'

Table S1. Primer sequences used in RT-qPCR.

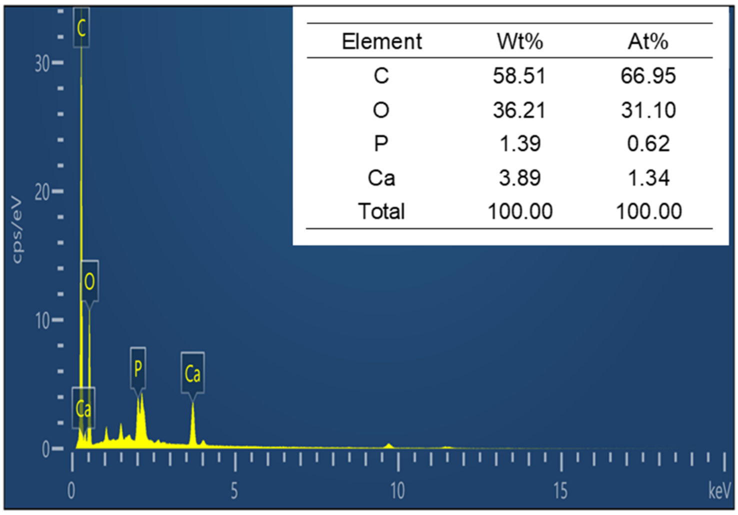

Figure S1. EDS spectrum with quantitative result.

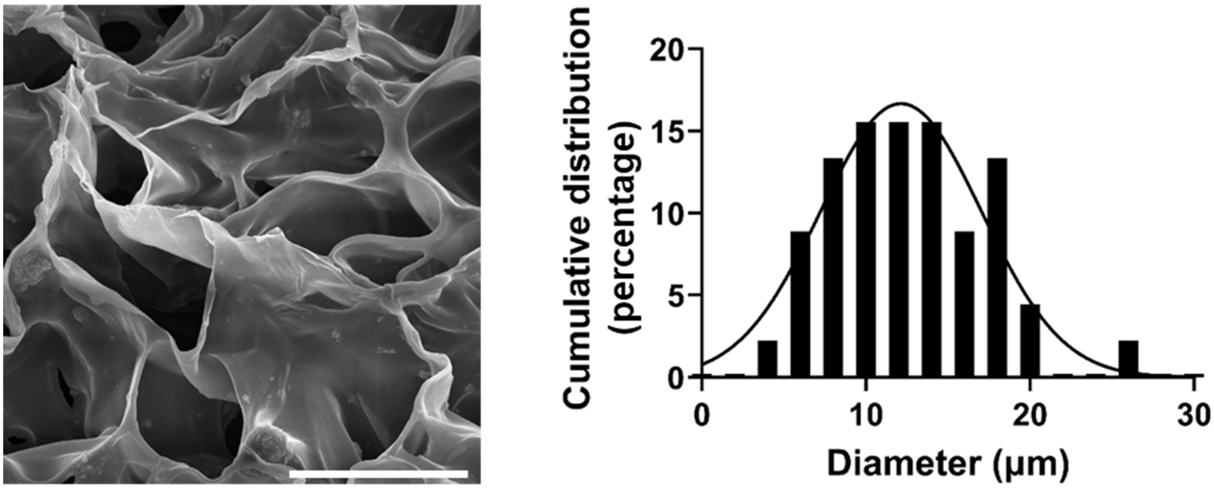

Figure S2. Pore size distribution of MSHA. Scale bar: 20 μm.

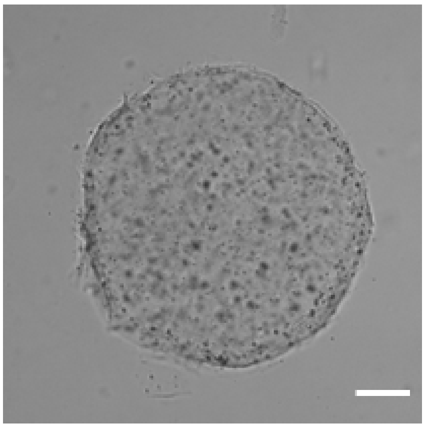

Figure S3. Optical microscopic image of MSHA after 7 days in an enzymatic environment. Scale bar:100 μm.

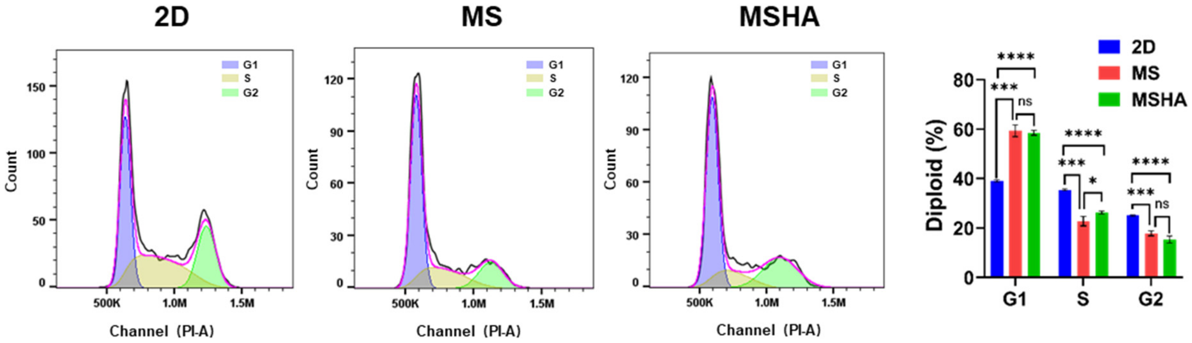

Figure S4. Cell cycle and quantitative results of MNNG/HOS cells by FC.

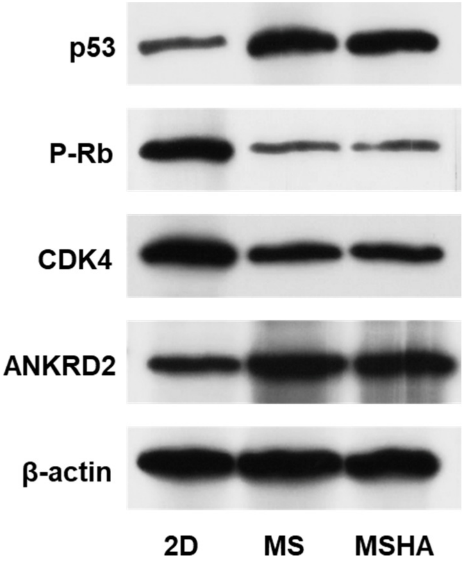

Figure S5. Protein expression levels of p53, Phospho-Rb (P-Rb), CDK4, and ANKRD2 in the 2D, MS, and MSHA groups.

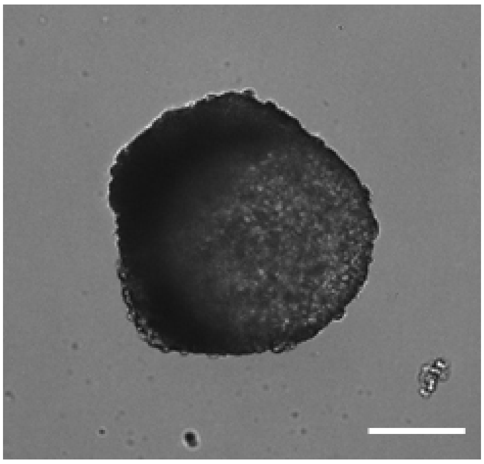

Figure S6. Optical microscopic image of a spheroid based on MSHA. Scale bar: 200 μm.

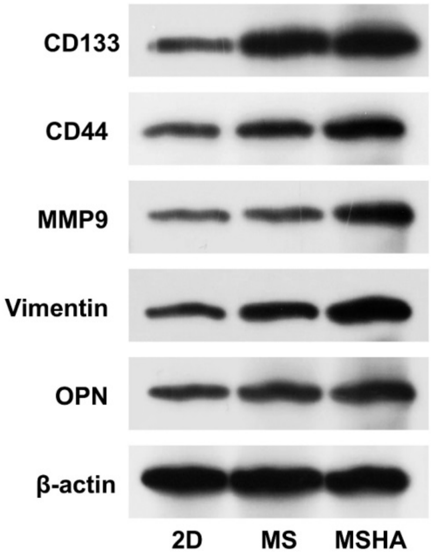

Figure S7. Protein expression of CD133, CD44, MMP9, Vimentin, and OPN, which are related to cell stemness, invasion, and migration.

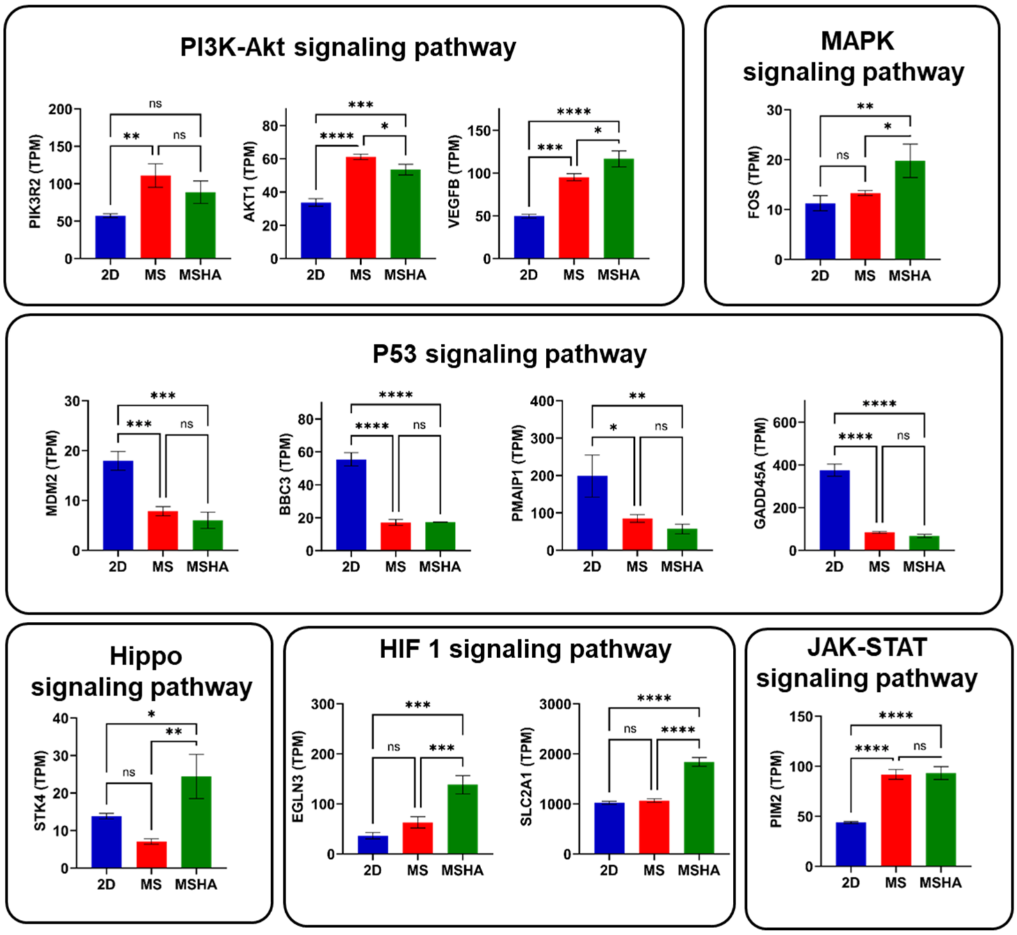

Figure S8. Expression of signaling transduction factors of pathways in cancer.

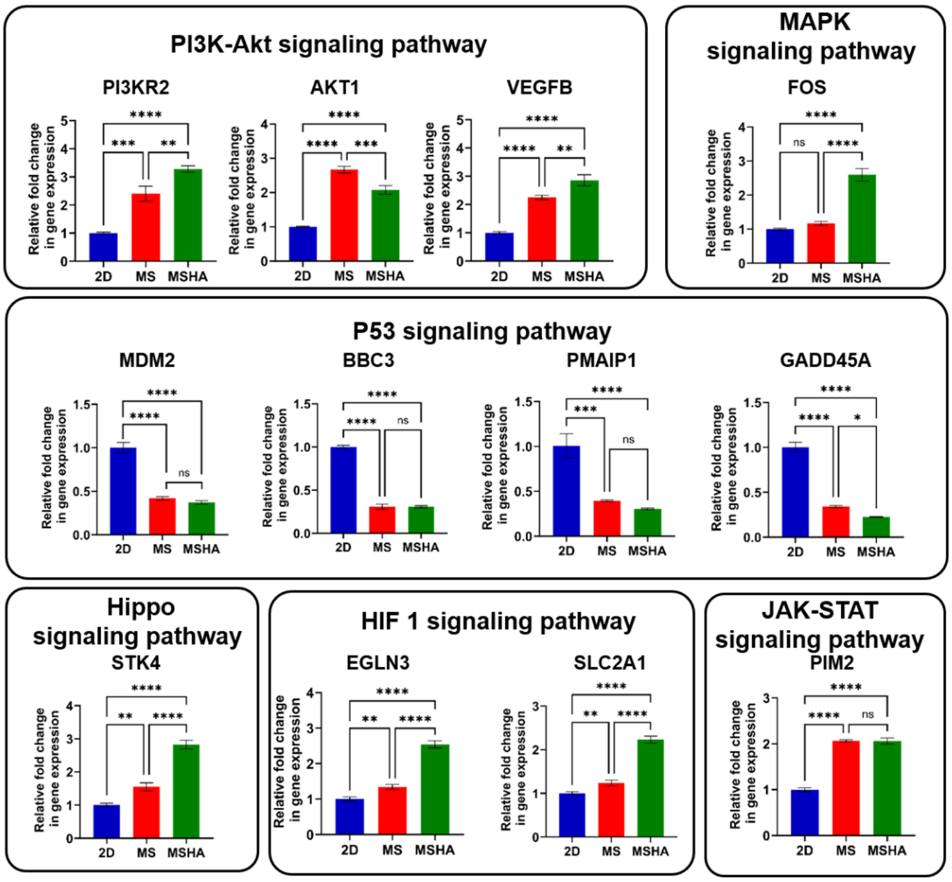

Figure S9. Gene expression of signal transduction factors involved in cancer-related pathways, as assessed by RT-qPCR.

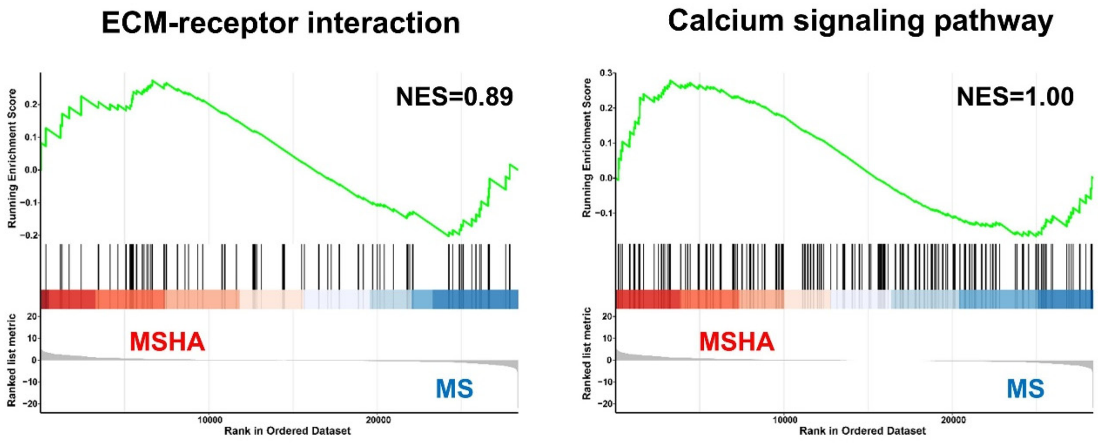

Figure S10. GSEA analysis of significantly changed pathways.

Reference

[1] He, J.; Chen, C.; Chen, L.; Cheng, R.; Sun, J.; Liu, X.; Wang, L.; Zhu, C.; Hu, S.; Xue, Y.; et al. Honeycomb-Like Hydrogel Microspheres for 3D Bulk Construction of Tumor Models. *Research (Wash D C)* **2022**, *2022*, 9809763.
[2] Wang, M. L.; Xu, N. Y.; Tang, R. Z.; Liu, X. Q. A 3D-printed scaffold-based osteosarcoma model allows to investigate tumor phenotypes and pathogenesis in an in vitro bone-mimicking niche. *Mater. Today Bio.* **2022**, *15*, 100295.
